# Impact of reducing portion sizes in worksite cafeterias: a stepped wedge randomised controlled pilot trial

**DOI:** 10.1186/s12966-018-0705-1

**Published:** 2018-08-16

**Authors:** Gareth J. Hollands, Emma Cartwright, Mark Pilling, Rachel Pechey, Milica Vasiljevic, Susan A. Jebb, Theresa M. Marteau

**Affiliations:** 1 Behaviour and Health Research Unit, University of Cambridge, Cambridge, UK; 2 Nuffield Department of Primary Care Health Sciences, University of Oxford, Oxford, UK

**Keywords:** Portion size, Workplace interventions, Physical micro-environment, Choice architecture, Nudging, Stepped wedge trial, Randomised controlled trial

## Abstract

**Background:**

Reducing the portion sizes of foods available in restaurants and cafeterias is one promising approach to reducing energy intake, but there is little evidence of its impact from randomised studies in field settings. This study aims to *i.* examine the feasibility and acceptability, and *ii.* estimate the impact on energy purchased, of reducing portion sizes in worksite cafeterias.

**Methods:**

Nine worksites in England were recruited to reduce by at least 10% the portion sizes of foods available in their cafeterias from targeted categories (main meals, sides, desserts, cakes). In a stepped wedge randomised controlled pilot trial, each site was randomised to a date of implementation, staggered fortnightly, following a baseline period of four weeks. Impact on energy purchased was analysed using generalised linear mixed modelling. We also assessed feasibility, acceptability, and fidelity of intervention implementation.

**Results:**

Data from six of the nine randomised sites were analysed, with three sites excluded for not providing sufficient data and/or not implementing the intervention. The extent to which the intervention was implemented varied by site, with between 6 and 49% of products altered within targeted categories. Feedback following the intervention suggested it was broadly acceptable to customers and cafeteria staff. For the primary outcome of daily energy (kcal) purchased from intervention categories, there was no statistically significant change when data from all six sites were pooled: percentage change − 8.9% (95% CI: -16.7, − 0.4; *p* = 0.081). Each of these six sites showed reductions in energy purchased, ranging from − 15.6 to − 0.3%, which were borderline statistically significant at two sites (respective percentage changes (95% CIs): − 15.6% (− 26.7, − 2.8); − 14.0% (− 25.0, − 1.2)). Secondary outcome data are suggestive of a compensatory increase in energy purchased from food categories not targeted by the intervention, with no overall effect observed on energy purchased across all categories.

**Conclusions:**

The results of this pilot trial suggest that reducing portion sizes could be effective in reducing energy purchased and consumed from targeted food categories, and merits investigation in a larger trial. Future studies will need to address factors that prevented optimal implementation including site dropout and application across a limited range of products.

**Trial Registration:**

(ISRCTN52923504). Registered on 20th September 2016.

**Electronic supplementary material:**

The online version of this article (10.1186/s12966-018-0705-1) contains supplementary material, which is available to authorized users.

## Introduction

Unhealthy patterns of food consumption, including excess energy intake, make a major contribution to the burden of non-communicable disease that accounts for more than two-thirds of deaths worldwide [1, 2]. The immediate physical environments with which we interact exert a considerable influence on selection and consumption of food, potentially without awareness [3–5]. As such, changing cues within physical micro-environments such as restaurants and shops could act as a catalyst for changing behaviour to improve health [6–9]. Worksite cafeterias are a particularly important intervention setting, with estimates suggesting that at least one-third of an adult’s daily energy intake is consumed when at work [10]. A key environmental cue to consumption, and therefore a promising target for interventions in physical micro-environments, is the portion size of foods available for selection, purchase and consumption. This is supported by the findings of a Cochrane review [11] as well as other systematic and narrative reviews [12–14] that consistently highlight the important effect of portion size on consumption.

A key limitation of the existing evidence base is that most studies that attempt to isolate the singular effect of reducing portion sizes have been conducted in controlled, laboratory settings [11] with few studies in field settings, especially involving adults. Studies in workplaces have typically tested the impact of multiple concurrent intervention components [15, 16], while studies of adults in uncontrolled restaurant or cafeteria settings are scarce and typically make few reductions in portion size across the range of available food options, and across few sites [17, 18]. A small number of studies have implemented a promising but slightly different intervention in which reduced portion options are added to the range of available options, although with larger sizes remaining available [19–21].

The current study instead focuses on an intervention that effectively removes larger portion sizes from sale and replaces them with reduced sizes of the same product. A recent study [22] used this approach to reduce portions of meat in three restaurants using a randomised crossover design. However, this was only in some main meal selections and was combined with simultaneously increasing the portion sizes of accompanying vegetable servings. To our knowledge, the current study is the most comprehensive to date of an intervention to directly reduce portion sizes of menu items in a field setting, both in terms of the number of study sites, and the extent of the range of products targeted by the intervention.

Beyond estimating the potential behavioural impact of this intervention, the current study sought to examine the feasibility and acceptability of reducing portion size. Feasibility here concerns how readily this type of intervention can be implemented within a cafeteria setting, and an assessment of the feasibility of conducting a larger, potentially definitive, trial. Acceptability to key stakeholders, including the customers subject to the intervention, is another relevant concern given that this influences the likelihood of implementation by actors in positions to change environments. While there is evidence from general surveys that environmental interventions including limiting portion sizes are relatively acceptable to the public [23], there is more specific evidence from focus groups that portion size interventions may differ in their acceptability, with direct size reductions less likely to be accepted by consumers [24]. We are not, however, aware of intervention studies that have directly reduced portion sizes in this way and examined feasibility and acceptability within the same setting.

## Methods

### Design

A stepped wedge randomised controlled trial design was used (*for explanation of the trial design see* [25]), between March 2017 and July 2017. Following a baseline period of four weeks for all sites, each site was randomised to a date for implementing the intervention, staggered over six, two-week periods (steps), plus an additional one-week intervention period for all sites at the end of the trial. Two sites were randomised to each of the first three steps, with one site randomised to each of the final three steps (*see ‘Participating sites’ for further details*). This meant that the duration of the intervention at each site ranged from 3 to 13 weeks. Randomisation was conducted by the host research unit’s statistician using computer-generated random numbers. Following implementation, all sites were required to maintain the intervention until the end of the study. There was no allocation concealment as the research team enrolled and communicated with the sites and were therefore aware of their intervention assignment. Ethical approval for the study was obtained from the University of Cambridge Psychology Research Ethics Committee (Pre.2016.035).

### Participating sites

**Fig. 1:**
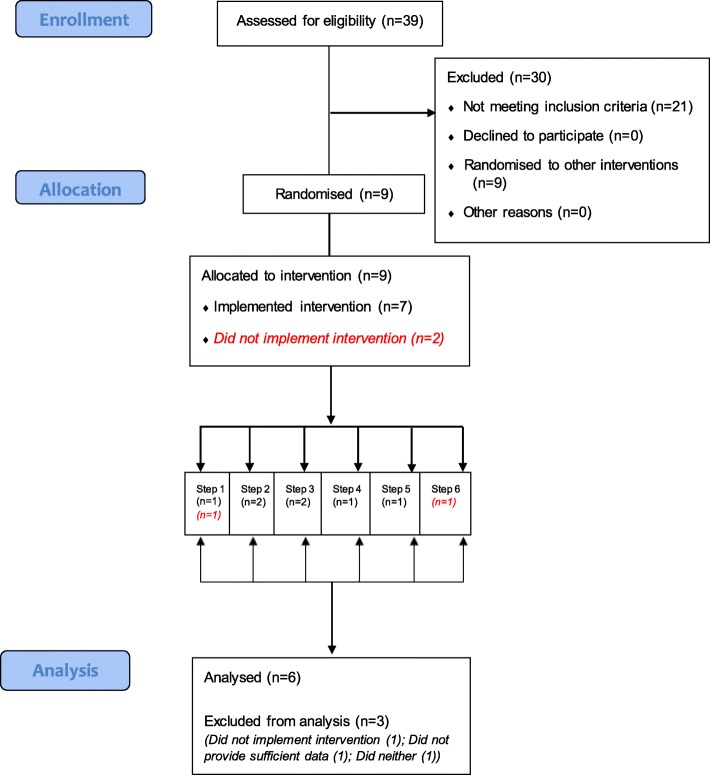
CONSORT Flow Diagram

Through a collaboration with IGD (Institute of Grocery Distribution; https://www.igd.com/), a charity set up to inform and educate the food and grocery industry about best practice, nine worksite cafeterias were recruited to the study (*see* Fig. 1 *for the CONSORT flow diagram*). The number of sites for this pilot study was determined on the pragmatic basis of maximising sample size given available resources. IGD invited managers of worksite cafeterias that: (a) were located in England, (b) had approximately ≥350 employees, and (c) could provide at least weekly sales data on individual items and the energy (kcal) content of items sold. Sites that volunteered were selected to include both office-based and depot/manufacturing sites. Due to the pilot nature of this trial, one site (Site 6) was recruited despite having fewer than 350 employees. The current study was conducted as one of a set of three separate intervention trials in worksite cafeterias, with the other two pilot trials focusing on a nutritional labelling intervention [26] and an intervention to alter the proportion of healthier vs less healthy foods (Pechey et al., under review). As such, the nine worksites that were initially recruited were identified from a pool of 39 worksites that had expressed an interest in participating in research in their cafeterias. Three had previously participated in the aforementioned nutritional labelling intervention and had continued with providing nutritional labelling in their cafeterias. One of these sites was randomised to each of the first three steps (of six total steps). The other six sites had not previously participated in any intervention and one of these sites was randomised to each of the six steps.

### Intervention

The intervention comprised reducing the portion sizes (by volume without changing energy density) of specified food items that were available in the worksite cafeterias (a Size x Product intervention within the TIPPME intervention typology [7]). It was also required that items reduced in size were priced proportionately to the reduction so that value-for-money remained consistent, and that the range of available food products was unchanged. The intervention targeted four product categories that initial consultation with cafeteria managers had suggested were most feasible for intervention, being main meals, sides, desserts, and cakes. Within these categories, changes were specifically requested for (but not limited to) all products that were trayed (e.g. pies), countable in pieces (e.g. scampi), wet/served with a ladle (e.g. curry, rice) or sliced or portioned by the sites (e.g. cakes), as these allowed reductions to be made most readily and precisely.

The four intervention categories (main meals, sides, desserts, cakes) were defined as follows:

#### Main meals

The meat or vegetarian principal element of a meal. In Site 3 only, this did not include any accompanying sides (e.g. rice).

#### Sides

Carbohydrate-rich portions (e.g. chips), vegetable portions (e.g. peas), and protein pots (e.g. tuna, cheese). This did not include those where they were provided as part of main meals.

#### Desserts

Hot desserts (e.g. crumbles), yoghurts, ice creams, and dessert pots (e.g. cheesecake, mousse, jelly, granola).

#### Cakes

Slices of cake and tray-bakes.

Categories that were not targeted by the intervention were those that cafeteria managers had indicated were not viable to intervene upon, such as pre-packaged foods (which were not targeted due to sites typically already selling the smallest commercially available sizes) and items for which the customer pays relative to the quantity they self-serve (e.g. salads). In total, these non-intervention categories comprised breakfast foods, hot and cold drinks, soups, savoury snacks, sweet snacks, sandwiches and wraps, salads and self-serve foods, and fruit.

### Procedure

A list of products available in each cafeteria was obtained from each site upon recruitment, and a site visit was conducted to explain the trial procedures and identify and resolve any initial issues for intervention implementation. The research team identified specific products that could be targeted, with the catering management making the final decision on what was intervened on. The reductions in portion size varied by site and specific product but were requested to be at least a 10% reduction by volume in each targeted product. Along with keeping a consistent range of available products throughout the duration of the study period, sites were also requested to maintain a consistent environment in other respects, such as cafeteria decoration, pricing of products outwith the intervention, and promotion and marketing activities.

Within the first intervention week at each site, compliance visits were conducted in which researchers visited the cafeteria to ensure agreed changes had been made (with photographs of intervention items sent by the sites on a weekly basis after that to enable corroboration). Cafeteria customers were not informed of the portion size reductions or commensurate price reductions. In sites where it was anticipated customers would notice and query reductions, staff (who were necessarily not blinded to the intervention) were instructed to respond to any queries from customers with a non-specific response concerning management trying various changes to the range of available food. In one site (Site 3) the cafeteria chose to inform customers that some products would be reduced in size. Cafeteria staff were supervised by cafeteria management throughout the study, and did not receive any direct training from the research team. Data on the energy content of each item sold in the cafeteria were provided by each site, with daily sales data obtained from the till records of each site.

### Measures

#### Feasibility and acceptability

These assessments are detailed in the protocol [25] and summarised as follows:

1. Feasibility of recruiting and retaining eligible worksites in a trial: assessed by recruitment and drop-out rates.
2. Feasibility of implementing the intervention: assessed after initial visits to worksite cafeterias by the research team, in discussions and formal interviews with worksite managers and catering teams, and through examination of the sites’ sales data.
3. Acceptability of the intervention: measured by surveying cafeteria customers and summarised with descriptive statistics, complemented by qualitative interviews with worksite or catering managers.
4. Compliance with the study protocol: assessed during compliance visits conducted during the first week following intervention implementation for each worksite, and via photographs of intervention items that were sent by the sites each week thereafter.

#### Intervention impact on consumption

Primary outcome:

- Total energy (kcal) purchased per day from intervention categories.

Secondary outcomes:

- Total energy (kcal) purchased per day from non-intervention categories.
- Total energy (kcal) purchased per day from all categories (for all items with calorie information).

Covariates:

- Total number of transactions per day from all categories, to account for busyness.
- Day of the week, given regular within-week fluctuations in sales.
- Number of days pre−/post-intervention, to allow for time trends.
- Daily mean temperature, daily hours of sunshine, and daily rainfall in site location, to account for changing weather conditions that may impact upon sales patterns.

#### Changes from published protocol

Since the publication of the protocol for this study [25], the primary outcome has been limited to the energy purchased from targeted intervention categories only. At the time of discussing potential changes with cafeteria management, it was clear that it was only feasible to intervene within certain product categories, with many categories (such as pre-packaged foods) unable to be changed. The registered primary outcome was changed to reflect this (registered at http://www.isrctn.com/ISRCTN52923504 on 31/5/2017), prior to completion of data collection and prior to any inspection of the data. At this point, the original primary outcome of total energy purchased per day from all categories became a specified secondary outcome.

### Analysis of intervention impact

Prior to any data analysis, any sites that did not provide sufficient data (we required sales data for the duration of the study period that identified purchases at the individual product level), or that had not implemented the planned intervention as previously described, were excluded from subsequent analysis. Energy (calorie) information provided by sites was matched to their sales data. Where sales of different product items had been recorded using the same till button, the median energy value of items recorded under the till button was used. Items with no energy information available across the study period were treated as missing. Daily sales values that were possible statistical outliers based on visual inspection of data plots were investigated by discussing with the sites whether there were any unusual circumstances that could explain them. Sites could not identify reasons that any data should be treated as invalid, and so they were generally considered to be part of the natural range of variation of such measurements. However, due to concerns about the unreasonable influence of any extreme and highly improbable values, we additionally used the median absolute deviation (MAD) approach to detecting statistical outliers for each analysis, applying a highly conservative threshold of 20 standard deviations. As a result, for the primary outcome analysis we excluded a single value representing one day’s sales at one site, being more than six times the median pre-intervention value at that site and which disproportionately influenced the effect estimates and worsened model fit. No values were excluded for analyses of secondary outcomes.

### Data analysis

Generalised linear mixed models in R version 3.4.2 with packages lme4 [27] and pbkrtest [28] were used. The daily energy purchased was logged in analyses due to skew in the data affecting the residuals in untransformed models. *P*-values were calculated using the Kenward-Roger method, to robustly adjust estimates for the small sample size relative to the number of explanatory variables. Primary outcome analysis was on the log of total energy (kcal) purchased per day from targeted food categories and examined the impact of the intervention (modelled using a dummy variable for intervention periods as randomised). In addition, the number of transactions, number of days pre-or post-intervention, and weather conditions (temperature, sunshine, rainfall) were modelled as fixed effects, with random effects for worksite (with day of the week nested therein as a random effect due to different daily patterns per site). The unit of analysis was the worksite cafeteria per day, as only aggregate till transaction data were available. To examine the impact of the intervention in each site separately, six separate dummy variables indicating the intervention period in each site replaced the overall intervention dummy variable in a follow-up analysis. Secondary outcomes were examined using the same analysis.

### Sensitivity analyses

As implementation of the intervention was delayed at two worksites, we pre-specified conducting sensitivity analyses to account for this. At Site 3, there was a two-week delay in the intervention being introduced. At Site 2, there was a one-week delay in the implementation of main meals and sides categories, which comprised the majority of the intervention items. As our main analysis treated intervention periods as randomised, a sensitivity analysis involved running the analyses with the intervention coded as starting from the date when the intervention was actually implemented at Sites 2 and 3. Given three sites had previously participated in a nutritional labelling intervention, we also conducted sensitivity analyses that accounted for the different pre-intervention comparator conditions by including a variable indicating whether each site had received no prior intervention or a labelling intervention.

## Results

### Feasibility and acceptability

#### Recruiting and retaining eligible worksites in a trial

All nine sites that were approached to take part agreed to participate in the study and were randomised. The flow of sites through the study is displayed in Fig. 1, with the characteristics of these sites displayed in Table 1. The type of sites varied, including office, depot and manufacturing sites, with this being reflected in the predominant occupational group. Of the nine randomised sites, three (33%) were not included in the analysis of intervention implementation and impact (although initial feasibility could be assessed); Site 7 did not implement the planned intervention, Site 8 neither implemented the intervention nor provided sufficient data, and Site 9 did not provide sufficient data (*see the following section for further details*). These results suggest that initial recruitment and randomisation of sites into a larger trial would be feasible, but that subsequent retention could be suboptimal.

**Table 1:** Characteristics of recruited sites

	Site								
	1	2	3	4	5	6	7	8	9
Type of site	Office	Manufacturing & Office	Depot	Office	Manufacturing	Manufacturing & Office	Depot	Manufacturing	Depot
No of employees	748	1154	668	956	477	269	1300	541	1060
Percentage that are full time	90.5	98.2	97.0	89.5	96.0	94.8	90.3	97.2	88.5
Mean age^a^	38.0	38.3	45.5	36.7	36.5	38.5	45.5	Missing	40.5
Percentage that are female	47.9	39.7	12.7	60.8	10.3	38.3	12.9	7.9	24.8
Predominant occupational group^b^	C1&C2	A&B	D&E	A&B	D&E	C1&C2	C1&C2	D&E	D&E
Mean cost of main meal (£)	3.25	3.34	2.75	3.25	2.83	3.00	1.20	2.53	1.28

^a^ Reported in age bands, and estimated using the mean age value for employees in each age band

^b^ A&B: Higher and intermediate managerial, administrative and professional occupations; C1&C2: Supervisory, clerical and junior managerial, administrative, professional occupations and skilled manual occupations; D&E: Semi-skilled and unskilled manual occupations

#### Feasibility of implementing the intervention

Once recruited, initial site visits and meetings with worksite managers and catering teams identified several important barriers to implementing the intervention in the nine randomised sites. These were grouped as follows:

#### Acceptability to cafeteria customers

In 6 of 9 sites, catering and worksite managers perceived that customers usually wanted the largest possible meal or meal with most calories, and so may be unhappy if they noticed that sizes had been reduced. This issue was explicitly reported by Site 3 as leading them to make only a small number of portion size reductions. Other sites appeared satisfied that it was manageable.

#### Proportionate pricing

In all 9 sites, catering and worksite managers emphasised the potential financial implications of the intervention. Caterers were often tied into contracts which would prevent proportional pricing, and some were also concerned that reducing portions could affect their profits. These issues were addressed by clarifying that senior management within the worksite companies had agreed to cover any additional costs introduced by the intervention for the study period.

#### Difficulty of implementation

In all 9 sites, catering and worksite managers emphasised that portion size reductions would be difficult to implement across the entire range of available products. For example, this was typically not possible for pre-packaged snack products or soft drinks as most cafeterias were already providing the smallest commercially available sizes. Other products were in theory possible to reduce in size but would place too much of a burden on staff, such as changing from self-served quantities of salads to having quantities measured out by staff, or ensuring that single pieces of meat or fish were reduced down in size, and were considered unviable. Relatedly, managers were concerned about the additional burden and workload placed on their staff having to change menus and working practices to reduce portions over the intervention period. Sites also did not all follow strict, pre-planned menus, making it difficult to clearly identify products in advance to be targeted by the intervention.

Through discussion between the research team and worksites, these issues were resolved sufficiently such that all nine randomised sites agreed to continue their participation and none dropped out of the study prior to the date at which they were due to introduce the intervention. However, two sites did not implement the planned intervention (Sites 7 and 8). Site 7 were not convinced that the intervention was feasible in its intended form, being concerned about perceived unacceptability to cafeteria patrons. As a result, they failed to implement the intervention by offering multiple different sizes simultaneously rather than replacing larger sizes with reduced sizes. Site 8 introduced a new cafeteria menu concurrent with the start of the intervention period, which while not explicitly linked to concerns about the intervention, highlights the difficulty for management in providing an otherwise unchanged cafeteria environment. Sites 8 and 9 did not provide sufficient data to enable inclusion in analysis because neither provided product-level sales data for the duration of the study period (Site 8 did not provide this for any weeks during the study period, while Site 9 provided this for only three study weeks and these data were incomplete). As a result of these issues at Sites 7, 8 and 9, data on the extent to which the intervention was implemented is derived from the remaining six sites.

Table 2 presents the intervention characteristics of the six sites. Main meals was the only targeted category where all sites implemented the intervention to at least some degree. Three sites intervened on sides, four intervened on desserts, and of the four sites that could have intervened on the cakes category (i.e. that sold products classed as cakes), only one did so. The extent of implementation of the intervention varied substantially by site, ranging from 5.6% of all available intervention category products at Site 3 to 49.4% at Site 6. It is also of note that the percentage of intervention category products that were changed relative to the number specifically targeted also varied, in particular, Sites 3 (15.5%) and 4 (12.4%) made very few changes, while Sites 6 (195.5%) and 1 (116.8%) made widespread changes beyond those initially suggested by the research team. This led to considerable variability; the size of main meals (reflected in mean calorie values at the category level) was reduced by an average of between 1.7% (Site 3) and 15.5% (Site 6). Reductions varied considerably by individual items within intervention categories, but were substantially greater for some items than the suggested minimum of 10%. For example, in Site 3, a portion of scampi was reduced from 514 to 386 kcal, resulting from a 25% decrease in the number of scampi pieces served as a main meal.

**Table 2:** Intervention characteristics by site

	Site					
	1	2	3	4	5	6
Proportion (%) of food/drink items for which energy content is available^a^	82.7	75.7	97.7	98.7	90.0	94.5
Proportion (%) of main meals targeted^b^ (targeted/all available)	38.3 (92/240)	30.6 (52/170)	26.9 (59/219)	68.7 (200/291)	52.0 (78/150)	24.2 (15/62)
Proportion (%) of main meals changed^c^ (changed/all available)	48.3^4^ (116/240)	30.6 (52/170)	9.1 (20/219)	9.6 (28/291)	23.3 (35/150)	62.9^4^ (39/62)
Mean kcal for a main meal pre-intervention; post-intervention (% diff.)	566; 522 (−7.8)	473; 444 (−6.1)	406; 399 (− 1.7)	381; 368 (− 3.4)	452; 443 (− 2.0)	446; 377 (− 15.5)
Proportion (%) of sides targeted^b^ (targeted/all available)	75.0 (6/8)	41.5 (27/65)	41.1 (44/107)	27.4 (23/84)	42.9 (9/21)	23.5 (4/17)
Proportion (%) of sides changed^c^ (changed/all available)	75.0 (6/8)	18.8 (12/65)	0 (0/107)	0 (0/84)	0 (0/21)	17.6 (2/17)
Mean kcal for a side pre-intervention; post-intervention (% diff.)	149; 137 (− 8.1)	180; 170 (− 5.6)	–	–	–	194; 183 (− 5.7)
Proportion (%) of desserts targeted^b^ (targeted/all available)	36.8 (7/19)	77.8 (7/9)	89.7 (26/29)	20.0 (1/5)	74.5 (35/47)	37.5 (3/8)
Proportion (%) of desserts changed^c^ (changed/all available)	10.5 (2/19)	11.1 (1/9)	0 (0/29)	0 (0/5)	70.0 (31/47)	25.0 (2/8)
Mean kcal for a dessert pre-intervention; post-intervention (% diff.)	154; 151 (− 1.9)	204; 192 (− 5.9)	–	–	401; 369 (− 8.0)	− 161; 148 (− 8.1)
Proportion (%) of cakes targeted^b^ (targeted/all available)	18.2 (2/11)	7.4 (2/27)	N/A^e^	33.3 (1/3)	9.1 (1/11)	N/A^e^
Proportion (%) of cakes changed^c^ (changed/all available)	9.1 (1/11)	0 (0/27)	–	0 (0/3)	0 (0/11)	–
Mean kcal for a cake pre-intervention; post-intervention (% diff.)	297; 286 (−3.7)	–	–	–	–	–
Proportion (%) of all intervention category items targeted (targeted/all available)^b^	38.5 (107/278)	32.5 (88/271)	36.3 (129/355)	58.7 (225/383)	53.7 (123/229)	25.3 (22/87)
Proportion (%) of all intervention category items changed (changed/all available)^c^	45.0 (125/278)	24.0 (65/271)	5.6 (20/355)	7.3 (28/383)	28.8 (66/229)	49.4 (43/87)
Mean kcal for all intervention category items pre-intervention; post-intervention (% diff.)	242; 230 (−5.0%)	285: 282 (−1.1%)	295; 287 (− 2.7%)	223; 222 (− 0.4%)	294; 284 (− 3.4%)	280; 250 (− 10.7%)
Proportion (%) of all targeted intervention items changed (changed/all targeted)	116.8^d^ (125/107)	73.9 (65/88)	15.5 (20/129)	12.4 (28/225)	53.7 (66/123)	195.5^d^ (43/22)

^a^Items where calorie information was not available were excluded from the study

^b^ Changes were requested across products that were trayed (e.g. pies), countable in pieces (e.g. scampi), wet/served with a ladle (e.g. curry, rice) or were sliced or portioned by the sites (e.g. cakes). Within these products, sites then agreed to make specific changes to menu items ^c^ Proportion of items during the whole intervention period (not including the baseline period) ^d^ Sites changed additional items to those requested ^e^No items within this category were on sale at these sites

#### Acceptability of the intervention

From the six sites that implemented the intervention, three sites (2, 3, and 6) conducted a post-intervention survey concerning acceptability of the intervention, with the other three sites not collecting feedback as they did not want to alert their customers that portion sizes had been reduced. Of the 2091 employees at the three sites, 175 customers (8.4%) responded to the survey. Two questions were asked of respondents. For the first question, “How did you feel about the portion size of products changing?”, 24% reported feeling pleased or very pleased, 19.4% felt neither pleased nor displeased, 29.1% felt displeased or very displeased, and 27.4% didn’t notice the changes. For the second question, “Would you like to see the changes remain in place permanently?”, 44% answered yes (either “yes, definitely” or “yes, probably”), 22.3% didn’t mind, while 33.7% answered no (either “no, probably not” or “no, definitely not”). In summary, most respondents seemed to find the intervention acceptable or were indifferent to or unaware of its implementation, but a sizeable minority were not happy with the reductions in portion size and did not want to see them continue.

Table 3 highlights comments made by managers in the post-intervention interviews, grouped in the same way as for the barriers identified pre-intervention. Managers did not typically report many customer complaints, consistent with the customer survey suggesting most patrons were positive or indifferent to the changes. By contrast, the challenges of implementation were commonly highlighted, although responses indicated that these were reasonably surmountable.

**Table 3:** Manager comments relating to acceptability

***Acceptability to cafeteria customers*** “The intervention went quite smoothly really. A lot of the proposed issues that we thought may raise up hadn’t come to, didn’t come to anything. A lot of the people didn’t really notice a 10% variance in their portion sizing.” (Site 1) “I think at [site] the vast majority of people probably either won’t notice or it won’t really bother them sufficiently for them to say anything or do anything any different other than just go along with what’s there and available to them.” (Site 1) “So honestly there was no real negative feedback or issues regarding the people on site and this intervention happening” (Site 5) “I did expect people to notice that maybe their portion of lasagne was a bit smaller, the amount of pasta they got on their plate was a bit smaller but they actually haven’t so, or if they have they haven’t been concerned about it.” (Site 2 and 6) “There wasn’t any kind of ‘we want them bigger again’ or any fuss in any way so no I didn’t get any noise at all from anybody in terms of discontent” (Site 2 and 6) “I think with some of the colleagues there was some negative feedback. I think they thought it was another initiative from [catering provider] to make more money by reducing the portion sizes.” (Site 3) “I think because it was only a very minor change really that nobody seemed to come back and say it was an issue.” (Site 4)	
***Proportionate pricing*** “…the technical side gave us a little bit of a challenge reducing the pricing according to the dishes that we did because we didn’t do it across the whole board” (Site 1) “…didn’t really notice and then when they did once they realised that there was a price reduction they were kind of less bothered by it” (Site 5)	
***Difficulty of implementation*** “What we certainly didn’t anticipate up front when we were talking about these kind of experiments at a higher level is just how many complexities there would be around, I suppose when you then introduce the pricing factor as well” (Site 1 and 5) “…it’s just become difficult when you get down to the detail of how we actually make this work without upsetting our employees” (Site 1 and 5) “…this intervention and the portion sizes it’s been quite difficult to continue it because of the structure of the pricing and the contract” (Site 1 and 5) “We did think at the beginning when we talked about it that there could be a few issues…but it went, in real terms it was fine, no problem” (Site 5) “…the challenges were getting in the smaller size branded products, that don’t exist” (Site 2 and 6) “…we had the equipment on site so we already had some smaller ladles and cutting up the tray-bakes wasn’t an issue.” (Site 2 and 6) “…probably only looking at hot food, so the hot food counters, because a lot of the portion sizing on pre-packaged foods is pretty reasonable” (Site 4) “I think [they] struggled at the beginning to work out what they could reduce because a lot of our things we already have things that are relatively cut down. There wasn’t a huge amount of things that we could perhaps change in terms of pre-packaged things … they focused on the main meals where they could serve up slightly less of different dishes” (Site 4) “…quite a challenging one because we couldn’t advertise entirely what we were doing” (Site 1) “There’s this view that the guys working out of the factory need feeding up, they’ve got hard manual jobs haven’t they and obviously the definition of, of course they need a few more calories than perhaps someone who’s sitting at a desk all day” (Site 5 and 8) “…we just felt that actually seeing as how nobody had really noticed that we’d made these changes we didn’t want to draw their attention to it because we might feel that there would be a bit of a backlash” (Site 4)	

#### Compliance with the study protocol

In the six sites that implemented the intervention and provided data, compliance with the protocol was high. Once the portion size reductions were introduced, they were implemented consistently throughout the duration of the intervention period, although, as mentioned, implementation was delayed in two sites.

### Intervention impact

**Fig. 2:**
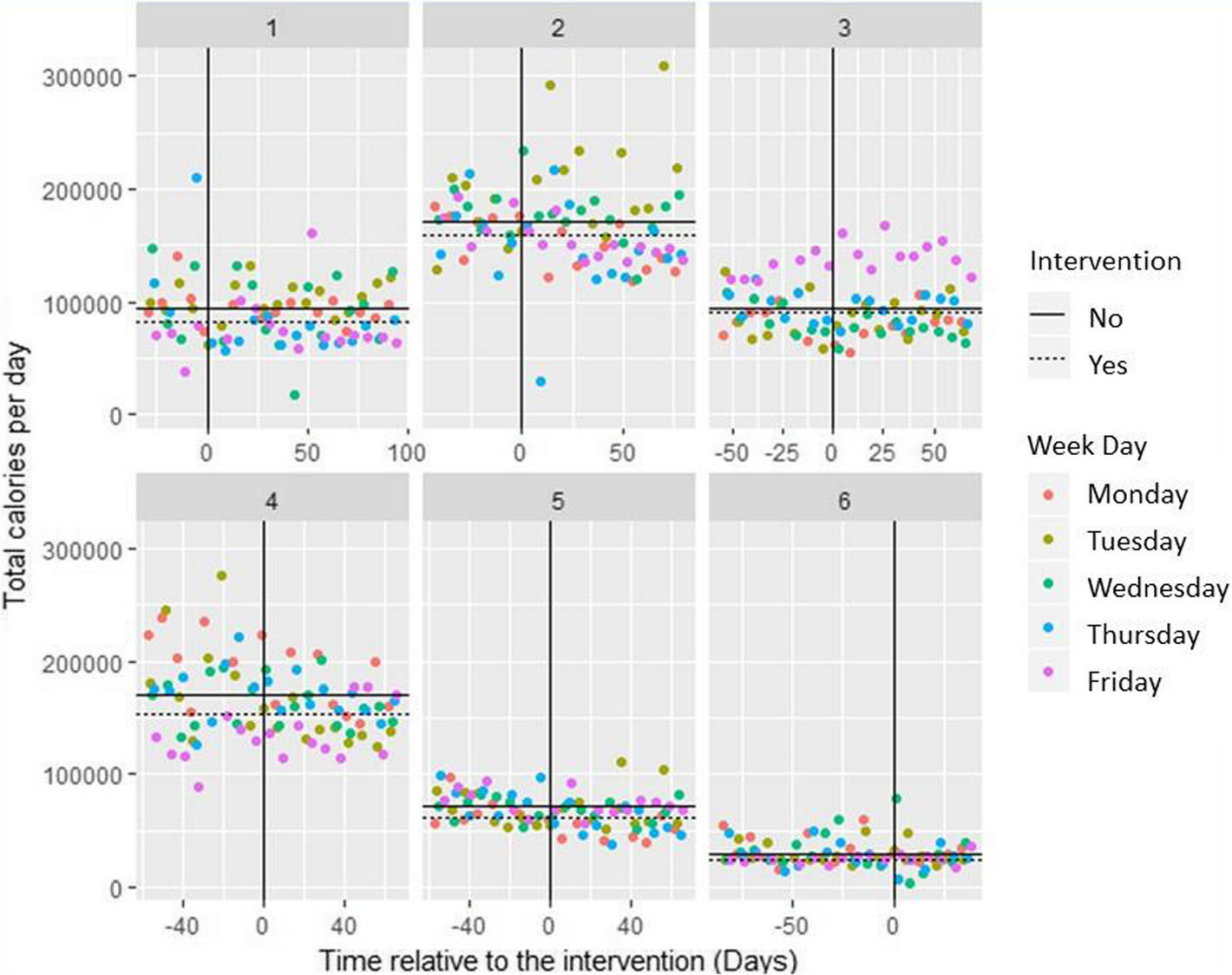
Scatterplots of daily energy purchased from targeted intervention categories over time, by site per day. Solid black lines indicate the geometric mean at each site pre-intervention; dotted lines post-intervention

Figure 2 shows the primary outcome measure of mean daily energy purchased by site from intervention categories. However, due to the different base levels of sales across sites and staggered implementation, a simple analysis of pre−/post-intervention means is not appropriate.

#### Primary outcome

For the primary outcome of daily energy (kcal) purchased from intervention categories, the mean effect of the intervention across all sites was a decrease of − 8.9% (95% CI: -16.7 to − 0.4; *p* = 0.081) which was not statistically significant. At the individual site level, each of the six sites showed reductions in energy purchased, ranging from − 15.6 to − 0.3%, which although not statistically significant at any site, were borderline significant at Sites 5 and 6. At Site 5, there was a mean reduction of − 14.0% (95% CI: -25.0 to − 1.2; *p* = 0.071), and at Site 6 there was a mean reduction of − 15.6% (95% CI: -26.7 to − 2.8); *p* = 0.051). Table 4 shows the results of analysis by site, with the logged coefficients back-transformed into percentage change (*See* Additional file 1: Tables S1 *and S2 for all model coefficients*). In the three sites where the greatest number of items in targeted categories were intervened on, being 49.4% at Site 6, 45% at Site 1 and 28.8% at Site 5, the largest intervention effects on the primary outcome were observed (respectively − 15.6, − 10.9% and − 14.0%). By contrast, Site 3, where the lowest proportion of intervention categories items was intervened on (5.6%), saw the weakest intervention effect of − 0.3%.

**Table 4:** Results of primary outcome analysis by site per day (log total calories)

Analysis	Variable		Coefficients (95% CIs)^a^	Percentage change (%) (95% CIs) ^a^	p^a^	Change in energy purchased (kcal)^b^	Mean number of transactions (s.d.)^b^	Change in energy purchased (kcal) per transaction^b^
Overall	Portion size intervention period		−0.093 (− 0.182, − 0.004)	−8.92 (− 16.68, − 0.44)	0.081	−10,208	537 (342)	−19.0
By site	Portion size intervention period	Site 1	−0.116 (− 0.271, 0.039)	− 10.94 (− 23.74, 4.01)	0.188	−12,541	460 (80)	−27.3
		Site 2	−0.052 (− 0.194, 0.090)	−5.05 (− 17.61, 9.44)	0.499	− 11,738	877 (129)	− 13.4
		Site 3	−0.003 (− 0.141, 0.134)	−0.32 (− 13.13, 14.38)	0.965	−2010	340 (28)	− 5.9
		Site 4	−0.074 (− 0.212, 0.064)	−7.13 (− 19.07, 6.56)	0.328	−15,575	1054 (233)	−14.8
		Site 5	−0.150 (− 0.288, − 0.012)	−13.96 (− 25.04, − 1.24)	0.071	− 10,034	338 (25)	−29.7
		Site 6	−0.170 (− 0.311, − 0.029)	−15.63 (− 26.74, − 2.84)	0.051	− 4468	147 (45)	−30.4

^a^ As the *p*-values (the more robust Kenward-Roger adjusted) and CIs (Wald) presented here have been calculated using different assumptions, there is not always an equivalence of interpretation between the 95% confidence intervals and significant p-values. ^b^ Based on raw data

#### Secondary outcomes

For the secondary outcome of daily energy purchased from non-intervention categories, the mean effect of the intervention across all sites was an increase of 7.3% (95% CI: 1.0 to 14.1; *p* = 0.067) which was not statistically significant. At the individual site level, each of the six sites showed increases in energy purchased, with effects ranging from 0.2 to 11.7%. None were statistically significant, although Sites 3 and 5 were borderline significant with respective, near-identical mean increases of 11.7% (95% CI: 1.6 to 22.8; *p* = 0.064) and 11.7% (95% CI: 1.6 to 22.8; *p* = 0.065). For the secondary outcome of total daily energy purchased from all food categories, the mean effect of the intervention across all sites was an increase of 0.4% (95% CI: -5.0 to 6.1, *p* = 0.890) which was not statistically significant. At the individual site level, three sites showed reductions in energy purchased, ranging from − 5.3% to − 1.3%, while three showed increases, ranging from 0.8 to 6.2%, none of which were statistically significant. See Table 5 for details of secondary outcomes.

**Table 5:** Regression coefficients and percentage changes for portion size intervention variables in analyses of secondary outcomes per day

Analysis	Variable		Purchasing from non-intervention categories. Coefficients (95% CIs)^a^	Purchasing from non-intervention categories. Percentage change (%) (95% CIs)^a^	p^a^	Total purchasing from all categories. Coefficients (95% CIs)^a^	Total purchasing from all categories. Percentage change (%) (95% CIs)^a^	p^a^
Overall	Intervention period		0.071 (0.010, 0.132)	7.321 (0.961, 14.083)	0.067	0.004 (− 0.051, 0.059)	0.408 (− 4.992, 6.116)	0.890
By site	Intervention period	Site 1	0.088 (−0.017,0.194)	9.242 (− 1.664, 21.358)	0.154	− 0.013 (− 0.110,0.083)	−1.338 (− 10.418, 8.663)	0.794
		Site 2	0.089 (−0.009, 0.186)	9.256 (− 0.904, 20.456)	0.129	0.035 (− 0.054, 0.123)	3.513 (− 5.227, 13.060)	0.474
		Site 3	0.111 (0.016, 0.205)	11.701 (1.631, 22.770)	0.064	0.060 (− 0.025, 0.146)	6.231 (− 2.465, 15.702)	0.218
		Site 4	0.028 (− 0.067, 0.122)	2.816 (− 6.455, 13.005)	0.587	−0.015 (− 0.100, 0.071)	−1.479 (− 9.546, 7.307)	0.745
		Site 5	0.111 (0.016, 0.205)	11.707 (1.608, 22.809)	0.065	0.008 (− 0.077, 0.094)	0.824 (− 7.451, 9.839)	0.858
		Site 6	0.002 (− 0.095, 0.099)	0.226 (−9.032, 10.426)	0.965	−0.054 (− 0.142, 0.034)	−5.257 (− 13.204, 3.418)	0.275

^a^ As the *p*-values (the more robust Kenward-Roger corrected) and CIs (Wald) presented here have been calculated using different assumptions, there is not always an equivalence of interpretation between the 95% confidence intervals and significant p-values

#### Sensitivity analyses

We conducted pre-specified sensitivity analyses accounting for delays in intervention implementation at Sites 2 and 3. Analysis of the primary and secondary outcomes was robust to using the actual intervention start date as opposed to intended start date and no conclusions changed. Analyses were also robust to accounting for whether sites had previously participated in a nutritional labelling intervention and again no conclusions changed.

## Discussion

The results of this study suggest that, first, a future trial of a portion size reduction intervention is feasible, with a high likelihood of initially recruiting and randomising worksite cafeterias. Significant barriers were identified in the randomised sites for implementing the planned intervention and providing necessary data. Nonetheless, six of nine sites in this pilot trial completed the study, suggesting that such barriers are often surmountable. Second, having been implemented, feedback following the intervention suggested it was broadly acceptable to customers and cafeteria staff. Third, the pattern of results suggests that portion size reductions could be effective in cutting energy purchased and thus consumed from targeted food categories. While this was a pilot rather than an appropriately powered confirmatory trial, and no effects were statistically significant in our primary analysis, there was sufficient evidence of feasibility and potential effectiveness to support a larger, potentially definitive, trial being conducted.

The direction and size of the effects at some worksites appears consistent with recent systematic review evidence showing that portion size reductions can substantially reduce selection and consumption of food [11, 12]. The variation observed between sites aligns with recent trials conducted in the same research programme that focus on nutritional labelling [26] and availability interventions (Pechey et al., under review), and previous results of interventions in physical micro-environments suggesting that effects may depend on context or intervention characteristics when applied within worksites [15] or more generally [29]. This inconsistency is unsurprising given the considerable variation in site characteristics in this study. For example, the cafeterias were within worksites that differed in size and function and related socio-demographic characteristics, with some being principally office-based with a more sedentary workforce from higher occupational groups, and others being depots or manufacturing plants with a physically active workforce. Worksite environments are also subject to considerable complexity, with an interplay of shifting physical, political, social and economic environments over time, at a multitude of levels including the cafeteria itself, the wider worksite and organisation that runs it, and the wider context, which all influence behaviour of both those intervening and those exposed to the intervention [30].

The observed reductions in energy purchased from intervention categories were apparent in spite of the widely varying, but generally modest, intervention fidelity. While we could not reasonably expect full implementation, four of six sites intervened on fewer than 30% of intervention category items. However, this suboptimal fidelity was not simply a function of sites being unwilling to apply the intervention, and may also have reflected an upper bound of what is practicable. It is notable that only 25–59% of intervention category items were specifically targeted by the intervention, this being determined by the types of products that were most feasible to reduce in size. While two sites intervened on fewer than 8% of intervention category products – clearly substantially fewer than were targeted - another two sites actually changed more items than were initially suggested by the research team. Constraints on intervening within intervention categories are also set within the wider context of these targeted categories only representing a subset of all the food and drink product categories available within cafeterias, with others considered unfeasible at the outset.

The importance of optimising the extent to which this intervention can be implemented is emphasised in light of the results for secondary outcomes. This suggests – albeit with the same due caution applied elsewhere about over-interpreting site-level data – that there may be a substantial compensatory increase in energy purchased from those categories that are not subject to portion size reductions, resulting in no overall effect on energy purchased across all categories. Clearly, should this finding be replicated, it has important implications for potential effectiveness. It suggests that to maximise effectiveness, impetus should be placed both on achieving wide implementation within each targeted category, and on extending implementation to a wider range of food categories. Realising these would minimise the capacity for substitution effects or compensatory purchasing, but where there are limited opportunities to do so, the current intervention would not be expected to have a meaningful effect on total energy consumed. Potential solutions involving extra staffing resources, more advanced administrative systems or changes to the available ranges of foods in cafeterias may therefore need to be considered and evaluated if implementation is to increase within categories currently targeted and be extended to additional categories.

### Strengths and limitations

To our knowledge, the current study is the most comprehensive to date of an intervention to directly reduce portion sizes of menu items in a field setting, in terms of the number of study sites, the breadth of products that were targeted, and the concurrent assessment of intervention feasibility and acceptability. The field nature of the cafeteria settings represents an important strength. While controlled laboratory studies provide valuable information on potential effects and underlying mechanisms, even when relatively naturalistic they are unable to provide insights into how people - importantly not just the recipients but also those involved in implementation - will respond within a complex food environment. Although challenging to develop and conduct, field studies are vitally important for building a conclusive evidence base for interventions.

The worksite settings also resulted in a number of study limitations. The absence of direct control of the cafeterias and their data collection systems likely impacted upon both intervention fidelity and data quality. Concerning intervention fidelity, while sites were instructed to refrain from introducing new promotional activities or making any physical e.g. decorative, changes to the cafeteria, it is possible that some (albeit likely minor) operational changes were made over the study period in at least some sites and not reported by cafeteria staff or identified by compliance checks. While working more closely with worksites and increasing levels of direct monitoring would require substantially more resources, it could enable increased confidence that this would be avoided. Regarding the quality of data, in some cases the data systems used by the cafeterias were basic and not ideal for this study. For example, we identified that a small number of items that cost the same were recorded on a single till button at some sites, with cafeteria management balancing the need to keep track of purchasing patterns with wishing to avoid the extra time and burden placed on their staff should they have to use more complex purchasing systems. A future trial would likely seek to recruit only sites that already used, or were willing to install, more advanced data systems capable of the detailed recording of sales data needed.

The certainty of the conclusions that can be drawn from this pilot trial are limited. Worksites were either unable, due to not having the necessary systems in place, or unwilling for ethical or privacy issues, to provide us with individual-level data for each customer, so the analysis used site-level data from only six cafeterias. This prevented examination of how individuals may have altered their purchasing behaviour in response to the intervention (such as buying additional servings or alternative products, or reducing their use of the cafeteria), and how this was modified by participant characteristics. Additionally, the variable nature of food purchasing meant that the data tended to be relatively disperse. This resulted in wide confidence intervals around effects, and the inability to map how individuals shifted their purchasing behaviour in response to the intervention. The lack of direct monitoring also meant that the veracity of specific data-points could not be independently corroborated or verified beyond the potentially fallible record-keeping and recall of cafeteria management. While there is no reason that these factors would have any systematic influence on the results, any conclusions based on estimating intervention impact from these data are necessarily cautious. Finally, although purchasing represents a strong and workable proxy for consumption, it is important to note that such outcomes are only indicative of potential impact on the amount of energy actually consumed. Again, with more resources, we could have attempted to model actual consumption in a more sophisticated manner, such as taking account of food waste at each site. It is also pertinent to note that while energy consumption in this context is an outcome that is indicative of likely health impact, we acknowledge that they are not synonymous – high energy food is typically nutritionally less healthy but is not always.

### Implications for research and policy

Given the burden of non-communicable disease [1, 2], there is a clear need for interventions to change behaviour and that have potential to be scaled up to population level, such as those that target physical micro-environments [7]. The results of this study extend prior evidence that reducing portion sizes may be a promising strategy to reduce energy purchased and in turn consumed. However, confirmatory or definitive trials are needed, likely with longer study durations and a larger number of worksites, as well as more intensive monitoring of, and support provided to, the implementation process. Additionally, more detailed process evaluations would be able to better identify related barriers and solutions.

In terms of policy implications, a key determinant of public health policy action, beyond simply providing sufficient evidence of effectiveness, is acceptability to key stakeholders including businesses and the publics targeted by such interventions. Encouragingly, the current study found that portion size reductions were relatively acceptable to cafeteria management and to patrons once they were implemented. Our findings are broadly consistent with those of an earlier focus group study of consumers [24] which whilst identifying some resistance to portion size reductions, found that there was general approval of interventions in this area and also of proportional pricing strategies (which were mandated in our intervention, but may not always be feasible). It should be noted, however, that customer feedback in the current study was provided by only a relatively small percentage of worksite employees and that this was provided in the context of modest intervention fidelity. We cannot assume similar support if the intervention were implemented more extensively, potentially increasing adverse responses in customers and cafeteria staff.

While there are key obstacles to introducing such interventions in commercial settings [31], there are likely fewer in quasi-commercial and public sector contexts [32]. The worksite cafeterias in the current study can be considered quasi-commercial because they cater to an on-site workforce and are not operated solely for profit. As such, should the worksite management wish to support changes to the cafeterias, commercial concerns may take less precedence than other concerns such as the health and wellbeing of the workforce. This aligns with previous findings [33] where representatives of worksite cafeterias were more receptive to interventions targeting portion size than those from standard commercial organisations. In public sector environments, food procurement plans for public bodies could include standard procurement of smaller portion sizes [34].

## Conclusion

The results of this pilot trial suggest that reducing portion sizes could be effective in reducing energy purchased and consumed from targeted food categories, and merits investigation in a larger trial. Future studies will need to address factors that prevented optimal implementation including site dropout and application across a limited range of products.

## Additional file

Additional file 1: **Table S1.** Regression coefficients (log total calories) and percentage changes from all variables in the primary outcome analysis aggregating across sites per day. **Table S2.** Regression coefficients (log total calories) and percentage changes from all variables in the primary outcome analysis by site per day. (DOCX 16 kb)
