# Supplementary material for: Impact of reducing portion sizes in worksite cafeterias: a stepped wedge randomised controlled pilot trial
Source: Int J Behav Nutr Phys Act. 2018 Aug 16;15:78. doi: 10.1186/s12966-018-0705-1 (PMC6097301; doi:10.1186/s12966-018-0705-1)
Supplement: Supplementary file 1 — Table S1. Regression coefficients (log total calories) and percentage changes from all variables in the primary outcome analysis aggregating across sites per day. Table S2. Regression coefficients (log total calories) and percentage changes from all variables in the primary outcome analysis by site per day. (DOCX 16 kb) [file 12966_2018_705_MOESM1_ESM.docx]

**Supplementary materials**

**Table S1. Regression coefficients (log total calories) and percentage changes from all variables in the primary outcome analysis aggregating across sites per day**

| **Variable** | **Coefficients**  **(95% CIs)^1^** | **Percentage change (%) (95% CIs)^1^** | **p^1^** |
| --- | --- | --- | --- |
| Portion size intervention | -0.0934  (-0.1825, -0.0044) | -8.92  (-16.68, -0.44) | 0.081 |
| Total number of transactions | 0.0004  (0.0002, 0.0006) | 0.04  (0.02, 0.06) | 0.015 |
| Number of days pre- or post-intervention^2^ | 0.0004  (-0.0011, 0.0017) | 0.04  (-0.09, 0.17) | 0.582 |
| Temperature (Celsius) | -0.0055  (-0.01407, 0.0031) | -0.55  (-1.40, 0.31) | 0.253 |
| Sunshine (hours) | -0.0007  (-0.0067, 0.0054) | -0.07  (-0.67, 0.54) | 0.837 |
| Rainfall (mm) | -0.0014  (-0.0069, 0.0041) | -0.14  (-0.69, 0.41) | 0.630 |
| *Intercept* | 11.2590  (10.7814, 11.7365) | - | <0.0001 |

^1^ As the p-values (the more robust Kenward-Roger corrected) and CIs (Wald) presented here have been calculated using different assumptions, there is not always an equivalence of interpretation between the 95% confidence intervals and significant p-values.

^2^ Day 0 is the day that the intervention was implemented

**Table S2. Regression coefficients (log total calories) and percentage changes from all variables in the primary outcome analysis by site per day**

| **Variable** | | **Coefficients**  **(95% CIs)^1^** | **Percentage change (%) (95% CIs)^1^** | **p^1^** |
| --- | --- | --- | --- | --- |
| Portion size intervention period | Site 1 | -0.1159  (-0.2711, 0.0393) | -10.94  (-23.74, 4.01) | 0.188 |
|  | Site 2 | -0.0518  (-0.1938, 0.0902) | -5.05  (-17.61, 9.44) | 0.499 |
|  | Site 3 | -0.0031  (-0.1407, 0.1343) | -0.32  (-13.13, 14.38) | 0.965 |
|  | Site 4 | -0.0740  (-0.2116, 0.0636) | -7.13  (-19.07, 6.56) | 0.328 |
|  | Site 5 | -0.1503  (-0.2882, -0.0124) | -13.96  (-25.04, -1.24) | 0.071 |
|  | Site 6 | -0.1700  (-0.3111, -0.0288) | -15.63  (-26.74, -2.84) | 0.052 |
| Total number of transactions | | 0.0004  (0.0002, 0.0007) | 0.04  (0.02, 0.07) | 0.013 |
| Number of days pre- or post-intervention^2^ | | 0.0004  (-0.0009, 0.0018) | 0.04  (-0.09, 0.18) | 0.539 |
| Temperature (Celsius) | | -0.0060  (-0.0146, 0.0026) | -0.60  (-1.45, 0.26) | 0.218 |
| Sunshine (hours) | | -0.0008  (-0.0069, 0.0052) | -0.08  (-0.69, 0.53) | 0.80 |
| Rainfall (mm) | | -0.0018  (-0.0074, 0.0037) | -0.18  (-0.73, 0.37) | 0.540 |
| *Intercept* | | 11.2586  (10.7971, 11.7200) | - | <0.0001 |

^1^ As the p-values (the more robust Kenward-Roger adjusted) and CIs (Wald) presented here have been calculated using different assumptions, there is not always an equivalence of interpretation between the 95% confidence intervals and significant p-values.

^2^ Day 0 is the day that the intervention was implemented
